# Anti-Inflammatory Effect and Cellular Uptake Mechanism of Carbon Nanodots in in Human Microvascular Endothelial Cells

**DOI:** 10.3390/nano11051247

**Published:** 2021-05-10

**Authors:** Sarah Belperain, Zi Yae Kang, Andrew Dunphy, Brandon Priebe, Norman H. L. Chiu, Zhenquan Jia

**Affiliations:** 1 Department of Biology, University of North Carolina at Greensboro, Greensboro, NC 27412, USA; srbelper@uncg.edu (S.B.); z_kang@uncg.edu (Z.Y.K.); amdunphy@uncg.edu (A.D.); bmpriebe@uncg.edu (B.P.); 2 Department of Chemistry and Biochemistry, University of North Carolina at Greensboro, Greensboro, NC 27412, USA; nhchiu@uncg.edu; 3 Department of Nanoscience, Joint School of Nanoscience and Nanoengineering, University of North Carolina at Greensboro, Greensboro, NC 27401, USA

**Keywords:** carbon nanodots, vascular inflammation, tumor necrosis factor-alpha

## Abstract

Cardiovascular disease (CVD) has become an increasingly important topic in the field of medical research due to the steadily increasing rates of mortality caused by this disease. With recent advancements in nanotechnology, a push for new, novel treatments for CVD utilizing these new materials has begun. Carbon Nanodots (CNDs), are a new form of nanoparticles that have been coveted due to the green synthesis method, biocompatibility, fluorescent capabilities and potential anti-antioxidant properties. With much research pouring into CNDs being used as bioimaging and drug delivery tools, few studies have been completed on their anti-inflammatory potential, especially in the cardiovascular system. CVD begins initially by endothelial cell inflammation. The cause of this inflammation can come from many sources; one being tumor necrosis factor (TNF-α), which can not only trigger inflammation but prolong its existence by causing a storm of pro-inflammatory cytokines. This study investigated the ability of CNDs to attenuate TNF-α induced inflammation in human microvascular endothelial cells (HMEC-1). Results show that CNDs at non-cytotoxic concentrations reduce the expression of pro-inflammatory genes, mainly Interleukin-8 (IL-8), and interleukin 1 beta (IL-1β). The uptake of CNDs by HMEC-1s was examined. Results from the studies involving channel blockers and endocytosis disruptors suggest that uptake takes place by endocytosis. These findings provide insights on the interaction CNDs and endothelial cells undergoing TNF-α induced cellular inflammation.

## 1. Introduction

Cardiovascular disease (CVD) has become a significant focus of research due to the impact and devastating effects it has within the world, and in the United States. Many risk factors of CVD have been associated with the formation of atherosclerosis, but inflammation and cellular dysfunction within the endothelial cell lining play one of the most significant roles in the initiation process [1,2,3,4]. This initiation causes the endothelial cells to signal an immune response by activating pathways that promote the expression of pro-inflammatory chemokines and cytokines such as tumor necrosis factor-alpha (TNF-α) and interleukin (IL)-8, interleukin 1 beta (IL-1β), as well as intercellular adhesion molecules (ICAM-1) [5,6,7,8].

Nanomedicine has made many advances within the biomedical community, and the treatment of many ailments using nanoparticles have been the direction many researchers have been taking. Through nanotechnology, these extremely small particles that can be less than 10 nm in size, known as carbon nanodots (CNDs), can be modified to fit the needs of the researcher, allowing to produce unique characteristics for each study [9]. These modifiable characteristics allow for expansive absorption and restricted emission ranges, along with outstanding electron donor and acceptor capabilities, and large surface area allowing for unique photoluminescent characteristics [10]. The photoluminescent characteristics of CNDs have been the primary focus, allowing biomedical researchers to use them in bioimaging and biosensing [10,11,12].

Due to the passivation surface of CNDs, they can be conjugated and doped with different dyes and stains, which enables biological imaging of cancer and immune cells [10,13]. Coupling these conjugating and doping capabilities with the extremely small size of CNDs, these dyes and stains can penetrate the cell membrane and even the nucleus with ease [10,14]. Not only is bioimaging possible by using these photoluminescent properties, but biosensing is as well. Due to the electron donor/accepting capabilities, CNDs can quench as well as renew their luminescence, allowing for the detection of materials such as Cu^2+^, H_2_O_2_ and glucose [11,15,16]. Bioimaging and biosensing have been researched extensively, and many groups are still probing these areas; however, some have changed their direction and are looking towards using CNDs as a drug delivery method.

With CNDs biocompatibility, drug delivery is another biomedical application research has been leaning towards. Synthesis of CNDs can be extremely simple, inexpensive materials to make, and the materials utilized to generates these particles can modify their properties and purposes [17]. Synthesizing CNDs out of pharmaceutical drugs to treat certain ailments has been one method in researching drug delivery techniques. Aspirin-based, as well as Metronidazole-based CNDs, have been synthesized to deliver these pharmaceuticals directly to the cells, allowing for lower dosages to be effective and efficient enough [14,18,19]. Extensive research has been completed in this area, along with the bioimaging and biosensing aspects of CNDs; however, the full capabilities and potential biomedical uses of CNDs have yet to be explored fully.

CNDs have been noted to be potent antioxidants and scavenging reactive oxygen species (ROS), and studies have been able to demonstrate protection against these free radicals as well as oxidative stress [20]. The mechanism behind these antioxidant properties is still unclear, but the oxidant-free radical scavenging capabilities are believed to be associated with the electron donor capabilities of CNDs. The large surface area of CNDs is made up of carboxyl, hydroxyl, and amino functional groups, which are believed to be responsible for the reduction and oxidation of these free radicals. The impact of CNDs antioxidant properties on the cardiovascular system has had limited exploration. TNF-α is known to trigger the pro-inflammatory response by stimulating the production of cytokines, chemokines, and adhesion molecules, causing cellular dysfunction within endothelial cells [21]. An increase in cellular ROS was reported to be involved in the inflammatory effects of TNF-α on endothelial cells [21]. However, CNDs effect on the vascular system, and their antioxidant properties’ role in anti-inflammation within vascular endothelial cells are still unclear. For this study, gene expression of pro-inflammatory cytokines, chemokines, adhesion molecules, and genes associated with phase II antioxidant enzymes were examined. The potential methods of CND entry within HMEC-1s were also completed in this study, by using the fluorescent properties of these nanoparticles along with certain known channel blockers and endocytosis disruptors.

## 2. Materials and Methods

### 2.1. Cell Culture

Human Microvascular Endothelial cells (ATCC^®^ CRL-3242™) cells were cultured in GenDepot^®^ MCDB131 media, supplemented with 10 mM L-glutamine, 10 ng/mL Endothelial Growth Factor (EGF), 1 g/mL hydrocortisone, 10% Fetal Bovine Serum (FBS), and 1% Penicillin/Streptomycin. Cells were grown in Cellstar Filtered Cap 75 cm^2^ cell-culture treated, filter screw cap flasks in an incubator set to 37 °C and 5% CO_2_. Media was renewed every 2–3 days, and cells were be split into a new passage at 85–90% confluence.

### 2.2. CND Synthesis and Characterization

The CND was synthesized and characterized based on our published microwave-assisted method [22,23,24]. CNDs were prepared by using citric acid (0.96 g, 99.5%, Sigma-Aldrich, St. Louis, MO, USA), ethylenediamine (1.0 mL, 99%, ACROS Organic, Fair Lawn, NJ, USA). This solution was mixed in a glass breaker and heated using a domestic microwave oven (1200 W) for 5.0 min. The resulting solution was then diluted with 10.0 mL deionized water and dialyzed using a 500–1000 Da MWCO (Spectra Por Float-A-Lyzer G2, Repligen, Waltham, MA, USA). Then, a lyophilizer (Labconco FreeZone Plus 12, Labconco Corporation, Kansas City, MO, USA) was applied to dry the solution to obtain the final solid product. The Cary^®^ Eclipse™ Fluorescence Spectrophotometer was used for UV–VIS spectroscopy. CNDs were diluted to a 2 mg/mL concentration in deionized water, and the fluorescence properties were measured in a quartz cuvette.

### 2.3. CND and TNF-α Treatments

Human Microvascular Endothelial cells (HMEC-1) were treated with 10 ng/mL of TNF-α for 6 h with a dose-dependent co-treatment of 0.03, 0.1 and 0.3 mg/mL CNDs in Sigma-Aldrich/Millipore Sigma^®^ Hanks Balanced Salt Solution (HBSS) containing calcium, magnesium, and glucose. Cells were split into 100 mm × 10 mm Corning^®^ Cell Culture-treated petri-dishes and grown to 85–90% confluence. Petri dishes were decanted of their media and rinsed 2 times with 1× Phosphate Buffer Solution (PBS). Treatment (7 mL) was then pipetted onto adherent HMEC-1s and maintained in an incubated at 37 °C and 5% CO_2_ during treatment.

### 2.4. CND Uptake Assay

HMEC-1 cells were cultured to 85% confluency in 100 mm × 10 mm Corning^®^ cell culture-treated Petri dishes. Cells were then washed 2 times with PBS and incubated for 30 min in HBSS and varying concentrations of known inhibitory reagents. After 30 min, cells were washed two times and incubated for 6 h with 0.1 mg/mL CNDs. Once 6 h treatment was completed, treatment was removed, and cells were washed 2 times with PBS to remove any excess CNDs. Cells were collected using the cell scrapping method, centrifuged at 5000 RCF for 5 min, and resuspended in 1 mL PBS. Then, 150 μL of resuspended cells was aliquoted into 4 wells on a black 96-well Costar^®^ plate. The fluorescence of the plate was then read using the Bio-Tek^®^ Synergy 2™ plate reader at 360/40 excitation and 460/40 emission.

### 2.5. Cell Viability with MTT Assay

HMEC-1 cells were grown in 24 well Costar^®^ cell culture-treated plate to 85–90% confluency. Media was removed, and cells were washed 2 times with PBS. Cells were then treated in 150 μL/well of HBSS with 0.001, 0.03, 0.1, 0.6 and 1.2 mg/mL of CNDs for 6 h. After treatment, cells were decanted and washed with 500 μL/well of PBS and then replaced with 0.2 g/mL MTT in 200 μL HBSS and incubated for 2 h. Following incubation, the MTT-mixture was removed, and cells were washed with PBS. Then, 200 μL of dimethyl sulfoxide (DMSO) was added to each well. The plate was covered and then placed on a shaker at low speed for 15 min. After purple formazan crystals that had formed were dissolved, cells were read with the Bio-Tek^®^ Synergy 2™ plate reader at 570 nm wavelength.

### 2.6. Measurements of IL-8 and sICAM-1 Protein Molecules

HMEC-1 cells were treated with 10 ng/mL of TNF-α in the presence or absence of 0.03, 0.1 or 0.3 mg/mL of CNDs for 6 h. IL-8 and soluble form of ICAM-1 (sICAM-1) in the cell culture supernatants were measured using Quantikine ELISA Kit (R&D Systems, Minneapolis, MN, USA) following the manufacturer’s instructions. Samples were plotted against standard curves for the determination of concentrations in the samples.

### 2.7. RNA Extraction

HMEC-1 cells were cultured within the 100 mm × 10 mm Corning^®^ cell culture-treated Petri dishes to 90% confluency. The media was discarded, and cells were washed with 1× PBS and treated in the TNF-α/CND concentrations previously described, for 6 h. After treatment, cells were decanted, and 1 mL of TRIzol^®^ (Ambion, Austin, TX, USA) was added to cells, and 1 mL of mixture was pipetted into a 1.5 mL Eppendorf tube. Then, 200 μL of Chloroform was added and incubated for 5 min at room temperature. Cells were then spun down in a centrifuge at 12,000 RCF for 15 min. The top aqueous layer was removed and combined with 500 μL of 2-propanol, mixed well, and incubated at room temperature for 15 min. The mixture was then centrifuged at 12,000 RCF for 10 min. Pellet formation was occurred and washed with 1 mL of 75% ethanol, centrifuged again at 7400 RCF for 5 min. The washing step was repeated once more. Pellet was then dried, and RNA was dissolved in 10 μL of Nase-free diethylpyrocarbonate (DEPC)-treated water.

### 2.8. cDNA Synthesis

HMEC-1 cells were cultured, treated, and RNA extraction was performed as listed above. The RNA extracted was quantified using Thermo Scientific NanoDrop™ and diluted to a 500 ng/μL concentration. Then 2 µL of diluted RNA were mixed with 5 μL of 5× First Strand Buffer, 1.25 μL of deoxynucleotide triphosphate (dNTP) solution, 1.25 μL of Random Primer, 0.625 μL of Moloney Murine Leukemia Virus Transcriptase (MMLV-RT), and 14.875 μL of DEPC water. The 25 µL solution was converted to cDNA using the Applied Biosystems™ Veriti™ 96-Well Thermal Cycler.

### 2.9. Quantitative Real Time-Polymerase Chain Reaction (qRT-PCR)

Cells were treated, RNA extracted, and cDNA synthesized as previously described. The cDNA was used to target genes of interest by combining 10 μL of Power SYBR^®^ Green PCR Master Mix, 5 μL of DEPC water, 2 μL of forward primer, and 2 μL of reverse primer for the genes of interest with 1 μL of the diluted (1:9) cDNA. The genes of interest were IL-8, ICAM, MCP-1, IL-1β, GCLC and HO-1 with GAPDH serving as the housekeeping gene. The Applied Biosystem^®^ StepOnePlus™ Real-Time PCR system ran for 40 cycles. Each cycle was as follows: 95 °C for 15 s, 58 °C phase for 1 min and a 60 °C cycle for 10 s. To quantify gene expression, the comparative threshold cycle (C_T_) values were used.

### 2.10. IDT^®^ Human Primer Sequences

The primer sequences are listed as followed: GAPDH Forward: 5′ AGA ACG GGA AGC TTG TCA TC-3′, GAPDH Reverse: 5′-GGA GGC ATT GCT GAT GAT CT-3′. IL-8 Forward: 5′ CTC TGT GTG AAG GTG CAG TT-3′, Reverse: 5′ AAA CTT CTC CAC AAC CCT CTG-3′. ICAM Forward: 5′ ACA GTG ACC ATC TAC AGC TTT C-3′, Reverse: 5′-CGG GTC TGG TTC TTG TGT ATA A-3′. MCP-1 Forward: 5′-GCT CAG CCA GAT GCA ATC AA-3′, Reverse: 5′-GGT TGT GGA GTG AGT GGT CAA G-3′. IL-1β Forward: 5′-CCA GCT ATG AAC TCC TTC TC-3′, Reverse: 5′-GCT TGT TCC TCA CAT CTC TC-3′. NQO-1 Forward: 5′-TTA

CTA TGG GAT GGG GTC CA-3′, NQO-1 Reverse: 5′-TCT CCC ATT TTT CAG

GCA AC-3′. GCLC Forward: 5′-ACC ATC ATC AAT GGG AAG GA-3′, GCLC

Reverse: 5′-GCG ATA AAC TCC CTC ATC CA-3′. AR Forward: 5′-TGG ATG GAT AGC TAC TCC GG-3′, Reverse: 5′-CCC AGA AGC TTC ATC TCC AC-3′.

### 2.11. Statistical Analysis

Statistical analyses were performed using GraphPad Prism^®^ software, and the data are expressed as mean ± SEM. Student *t* tests were performed to obtain statistical significance (*p* value). *p* < 0.05 was considered different.

## 3. Results

### 3.1. Characterization of CNDs: UV–VIS

**Figure 1:**
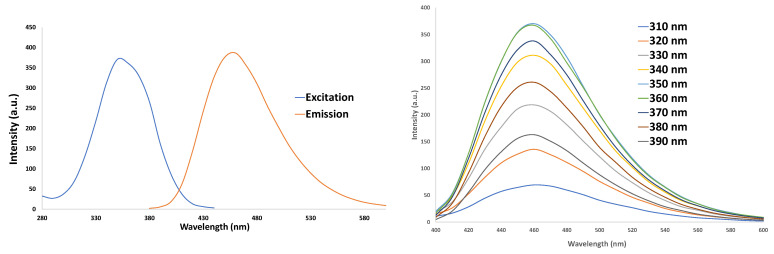
Characterization of CNDs. UV–VIS photoluminescence in a.u. Measurements taken by Cary^®^ Eclipse™ Fluorescence Spectrophotometer. Left panel: Absorption spectrum; Right panel: CNDs show emission peak of ~460 nm with excitation wavelengths from 400–600 nm.

The Cary^®^ Eclipse™ Fluorescence Spectrophotometer was used for characterizing the CNDs. The photoluminescent characteristics of CNDs can be seen in their excitation wavelength around 360 nm and expressed an emission peak around 460 nm (Figure 1). Understanding the excitation and emission spectra CNDs fall within allows for utilizing this information for the detection of the nanoparticles during uptake assays.

### 3.2. Cell Viability with MTT

**Figure 2:**
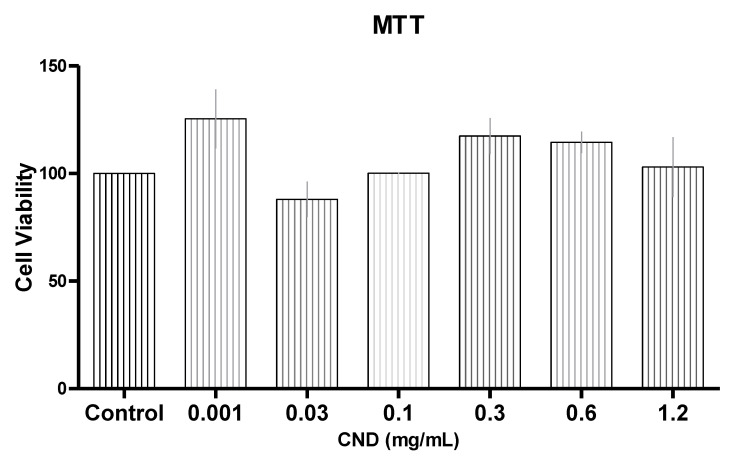
Cell viability with MTT assay. HMEC-1 cells were treated with varying concentrations of CNDs for 6 h. After treatment, cells were washed two times with PBS, and cells were treated with MTT dye for two hours. After two-hour incubation, purple formazan was dissolved in DMSO. Bio-Tek^®^ Synergy plate reader was used at 570 nm to determine colorimetric reading. No significant change in cell viability was determined to occur. All data represent mean ± SEM. (n = 3, *p* > 0.05 vs. control).

Before looking into potential anti-inflammatory properties of CNDs, it is important to determine if CNDs display any toxic effects to endothelial cells. A colorimetric assay known as an MTT assay was completed to analyze the mitochondrial function of HMEC-1 cells treated with 0.001, 0.03, 0.1, 0.3, 0.6, and 1.2 mg/mL of CNDs for 6 h (Figure 2). MTT is a positively charged tetrazolium compound that can easily penetrate viable eukaryotic cells [25]. CNDs at 0.001 mg/mL, 0.3 mg/mL and 0.6 mg/mL were found to have approximately 125%, 117%, and 114% of cell viability when compared to control, suggesting improved mitochondrial function with CND treatments. However, CNDs at all tested concentrations did not demonstrate any significant (*p* > 0.05) toxic effects when compared to the control, suggesting there is no significant change in mitochondrial function with CND treatments.

### 3.3. Quantitative Real Time-Polymerase Chain Reaction (qRT-PCR) for Proinflammatory Genes

We next wanted to determine the effect of CND’s on proinflammatory biomarkers in endothelial cells. HMEC-1 cells were treated with 0.03, 0.1, or 0.3 mg/mL of CND in the presence of 10 ng/mL of TNF-α for 6 h. After the 6 h treatment, cells underwent RNA extraction, cDNA synthesis, and qRT-PCR analysis. The dose concentrations were chosen based on our MTT cell viability assay (Figure 2) as well as our recent study [22].

**Figure 3:**
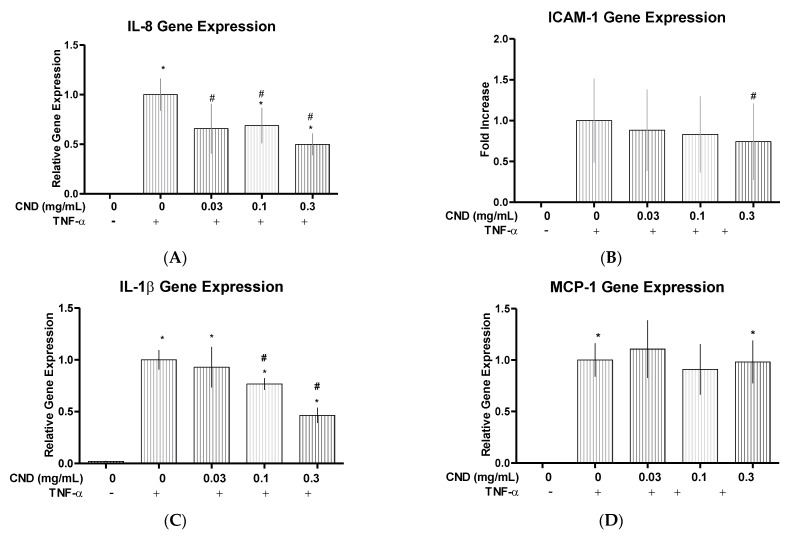
qRT-PCR proinflammatory gene expression. HMEC-1 cells underwent treatment with 10 ng/mL of TNF-α as indicated with the presence or absence of 0.03, 0.1, or 0.3 mg/mL of CNDs for 6 h. After treatment, cells underwent RNA extraction, cDNA synthesis, and qRT-PCR. (**A**) IL-8, (**B**) ICAM, (**C**) IL-1β, (**D**) MCP-1. A significant change was demonstrated during analysis of gene expression. All data represent mean ± SEM. (n = 3–6, * *p* < 0.05 vs. control, # *p* < 0.05 vs. TNF-α).

Our studies show IL-8 gene expression significantly decreased (*p* < 0.05), with cells that were co-treated with TNF-α and either 0.03, 0.1, and 0.3 mg/mL of CNDs, when compared to the cells treated with only TNF-α (Figure 3A). ICAM is another gene that is associated with proinflammatory cellular distress in endothelial cells. During our studies, when looking at ICAM gene expression during our treatment, the co-treatment of TNF-α with 0.3 mg/mL of CNDs displayed a significant decrease (*p* < 0.05), in gene expression when compared to cells treated with TNF-α alone (Figure 3B). We also found that cells treated with a co-treatment of TNF-α and either 0.1 or 0.3 mg/mL of CNDs had a significant decrease (*p* < 0.05), in gene expression of IL-1β, when compared to cells only treated with TNF-α (Figure 3C). Pro-inflammatory chemokine MCP-1 was also analyzed due to its important role in attracting monocytes to endothelial cells undergoing stress [26]. When analyzing MCP-1 with the qRT-PCR data, we found no significant difference in gene expression of any co-treated HMEC-1 cells (*p* > 0.05), when compared to cells treated with TNF-α (Figure 3D).

### 3.4. ELISA Assay for IL-8 and ICAM Protein Quantification

**Figure 4:**
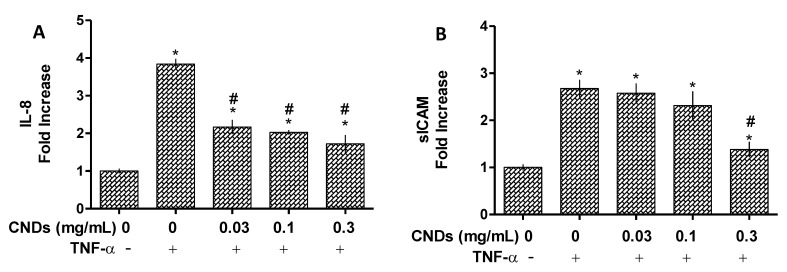
ELISA assay for IL-8 and ICAM protein quantification. HMEC-1 cells underwent treatment with 10 ng/mL of TNF-α as indicated with the presence or absence of 0.03, 0.1, or 0.3 mg/mL of CNDs for 6 h. After treatment, IL-8 (**A**) and soluble form of ICAM-1 (sICAM-1) (**B**) in the cell culture supernatants were measured by ELISA. All data represent mean ± SEM. (n = 3, * *p* < 0.05 vs. control; # *p* < 0.05 vs. TNF-α).

The effects of CNDs on IL-8 and ICAM at protein levels in HMEC-1 cells were examined by ELISA assay. As shown in Figure 4A, CNDs at 0.03, 0.1, and 0.3 mg/mL significantly inhibit TNF-α-induced the production of IL-8 in HMEC-1 cells (*p* < 0.05). Cells with a co-treatment of TNF-α and 0.3 mg/mL of CNDs also had a significant decrease (*p* < 0.05) in sICAM production induced by TNF-α when compared to the cells treated with TNF-α alone (Figure 4B). This inhibitory effect of CNDs on IL-8 and ICAM on the protein level detected by ELISA assay is consistent with its effect on the mRNA expression levels by qPCR assay (Figure 3).

### 3.5. Quantitative Real Time-Polymerase Chain Reaction (qRT-PCR) for ROS Detoxification Gene Expression

Excessive generation of ROS is known to cause inflammation, leading to endothelial dysfunction and damage. We wanted to see if the anti-inflammatory effects of CNDs are associated with their potential changes in expression of ROS detoxification genes such as NAD(P)H: Quinone Oxidoreductase 1 (NQO1), Nuclear factor erythroid 2 (Nrf2), aldose reductase (AR), Glutathione reductase (GR), Glutamate-Cysteine Ligase Catalytic Subunit (GCLC), and Heme Oxygenase 1 (HO-1). Treatment for HMEC-1 cells were followed as previously described, with a co-treatment of TNF-α and either 0.03, 0.1, or 0.3 mg/mL of CNDs for 6 h. After treatment, RNA extraction, cDNA synthesis, and qRT-PCR were completed.

**Figure 5:**
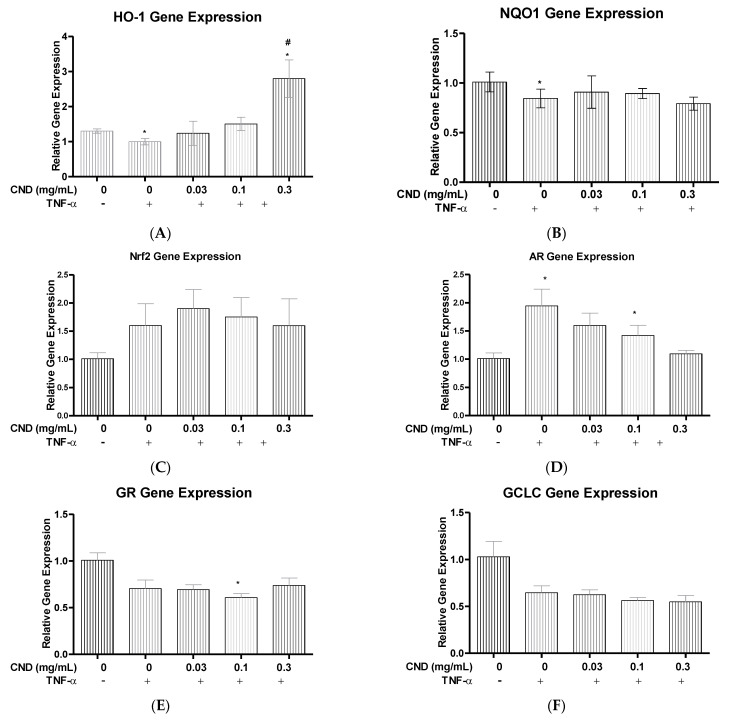
qRT-PCR Phase II antioxidant enzyme gene expression. HMEC-1 cells underwent treatment with 10 ng/mL of TNF-α as indicated with the presence or absence of 0.03, 0.1 or 0.3 mg/mL of CNDs for 6 h. After treatment, cells underwent RNA extraction, cDNA synthesis, and qRT-PCR. (**A**) HO-1, (**B**) NQO1, (**C**) Nrf2, (**D**) AR, (**E**) GR, (**F**) GCLC. Genes displayed no significant change in expression. All data represent mean ± SEM. (n = 3–6, * *p* < 0.05 vs. control, # *p* < 0.05 vs. TNF-α).

HO-1 is an important antioxidant that has the ability to protect endothelial cells from apoptosis. Previous studies have found that the overexpression of HO-1 can reduce TNF-α induced expression of proinflammatory genes such as E-selectin, ICAM, and Vascular adhesion molecule (VCAM), along with reducing the activation of the transcription factor, Nuclear Factor κ-light-chain enhancer (NF-κB) [27]. During our analysis, a significant increase in gene expression (*p* < 0.05) was seen for cells treated with a co-treatment of TNF-α and 0.3 mg/mL when compared to cells treated with only TNF-α (Figure 5A). NQO1 is another important antioxidant that is a quinone reductase, responsible for protecting cells from producing excess levels of ROS [28]. Our studies have shown that CNDs have no effect on NQO1 gene expression (*p* > 0.05), when comparing the co-treated TNF-α and CND cells with cells treated only with TNF-α (Figure 5B). Nrf2 is a transcription factor that can activate expression of promoters apart of the antioxidant response element (ARE), which are responsible for maintaining levels of ROS. Activation of Nrf2 has shown to be a potential method in reducing CVD [29]; however, our results demonstrate that CNDs have no significant effect on Nrf2 gene expression (*p* > 0.05), with co-treated cells compared to only TNF-α treated cells (Figure 5C). We wanted to see if CNDs had any effect on gene expression of another ROS modulator known as AR. Our results demonstrate that the co-treatment with TNF-α and CNDs had no significant change in gene expression (*p* > 0.05) of AR in HMEC-1s (Figure 5D). GR is another important antioxidant enzyme that catalyzes the reduction of glutathione disulfide (GSSG) to the sulfhydryl form glutathione (GSH) [30]. Previous studies have shown that GR knockout mice had an increase in proinflammatory cytokines such as TNF-α and IL-6 [31]. Results demonstrated no change (*p* > 0.05), in gene expression of GR comparing our co-treated HMEC-1s with cells treated only with TNF-α (Figure 5E).

### 3.6. CND Uptake Assay

**Figure 6:**
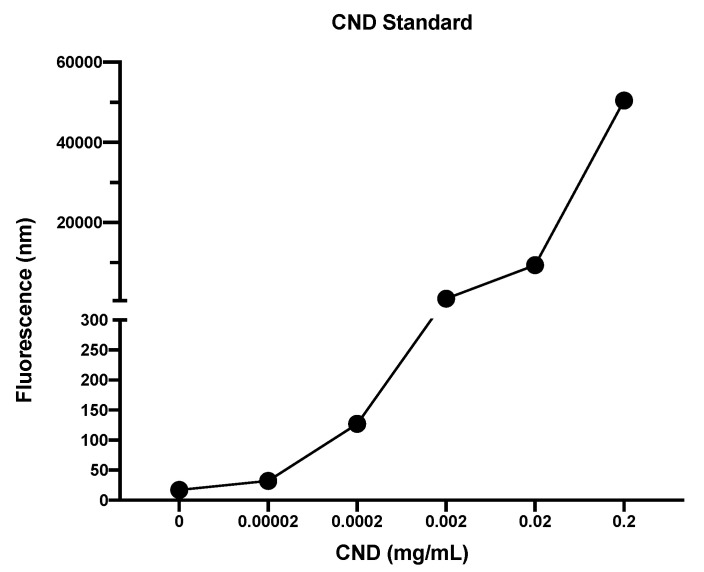
CND standard. A standard was created by completing a serial dilution of CNDS in di-water and then fluorescence was measured. Bio-Tek^®^ Synergy plate reader was used at 360/40 excitation and 460/40 emission.

**Figure 7:**
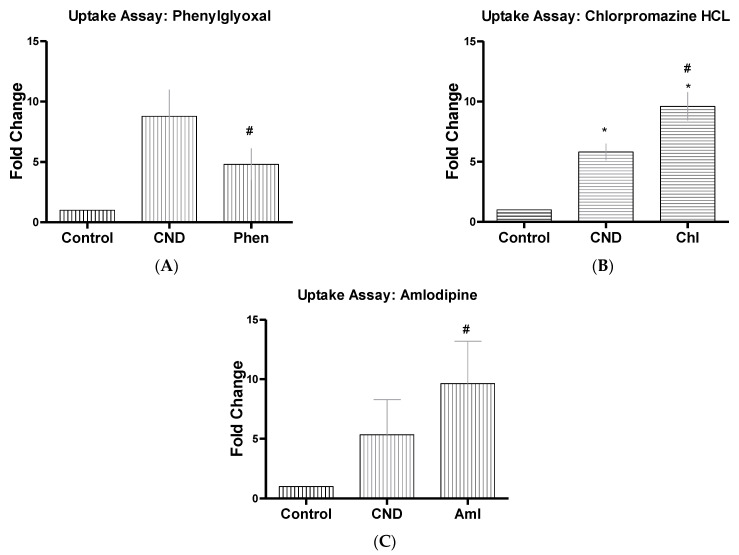
CND uptake. HMEC-1 cells were treated with inhibitor Phen (**A**), Chl (**B**) and Aml (**C**) with concentrations described (Table 1) for 30 min. Cells were then washed two times with PBS and treated for 6 h, with 0.1 mg/mL of CNDs. After CND treatment, cells were washed two times with PBS.

Determining the possible routes of entry of CNDs is important to understand the function and role these nanoparticles have in the cell. A standard curve of CNDs was created (Figure 6). Uptake had previously been studied in our lab using HMEC-1 cells, where cells were treated with 0, 0.03, 0.1, 0.2, and 0.3 mg/mL CNDs for either 6 or 12 h. Results displayed a dose-dependent increase in fluorescence for both the 6 and 12 h treatments. For determining possible route of entry within HMEC-1 cells, different inhibitors were tested individually to determine if any of them had the capability to reduce CND fluorescence. Endocytosis has been suggested to be the route of uptake for CNDs from previous research completed with different cell lines and different sized nanoparticles [32]. HMEC-1s were treated with endocytosis disrupting inhibitors for 30 min with concentrations determined from previous studies (Table 1). Inhibitors were removed, and cells were washed two times with 1× PBS and treated with 0.1 mg/mL of CNDs. Cells were then washed two times with 1× PBS to ensure any excess CNDs were removed. Using HMEC-1 cells showed a significant decrease (*p* < 0.05) in fluorescent intensity when cells were pre-treated with phenylglyoxal, which is a known selective phagocytic inhibitor (Figure 7A). Chlorpromazine HCL (Figure 7B), which is known to suppress clathrin disassembly, also had a significant change in fluorescence, showing a potential increase in CND uptake (*p* < 0.05).

**Figure 8:**
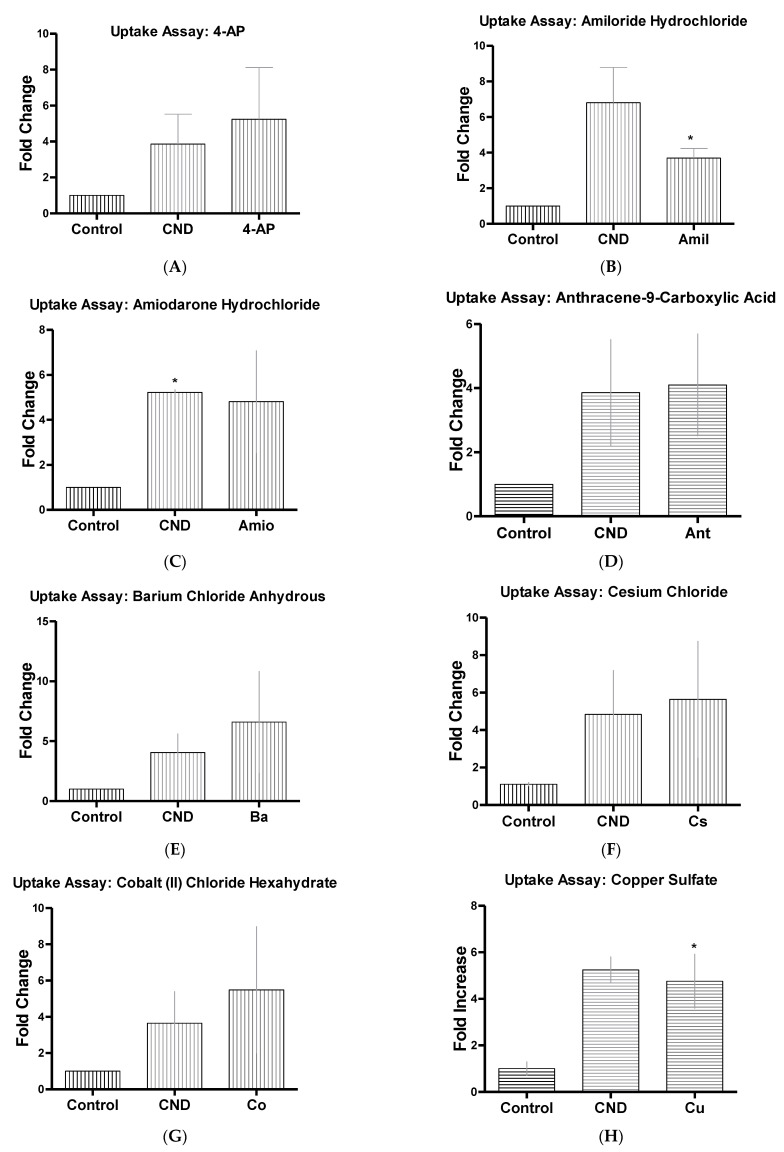

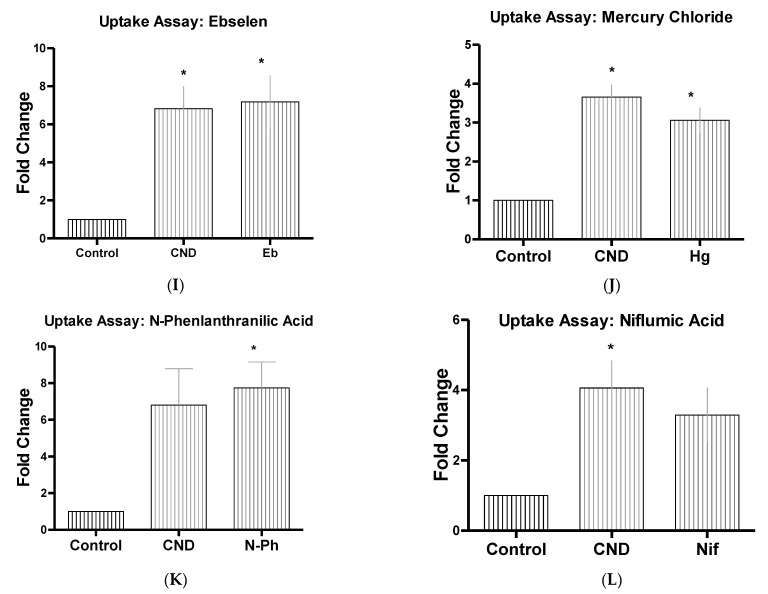
CND uptake with channel blockers HMEC-1 cells were treated with inhibitor 4-AP (**A**), Amil (**B**), Amio (**C**), Ant (**D**), Ba (**E**), Cs (**F**), Co (**G**), Cu (**H**),Eb (**I**), Hg (**J**), N-Ph (**K**), Nif (**L**) with concentrations described (Table 1), for 30 min. Cells were then washed two times with PBS and treated for 6 h with 0.1 mg/mL of CNDs. After CND treatment, cells were washed two times with PBS and removed. Cells’ fluorescence was checked using the Bio-Tek^®^ Synergy plate reader at 360/40 excitation and 460/40 emission. Channel blocking inhibitors displayed no significant change in fluorescent intensity. All data represent mean ± SEM. (n = 3–5, * *p* < 0.05 vs. Control).

**Figure 9:**
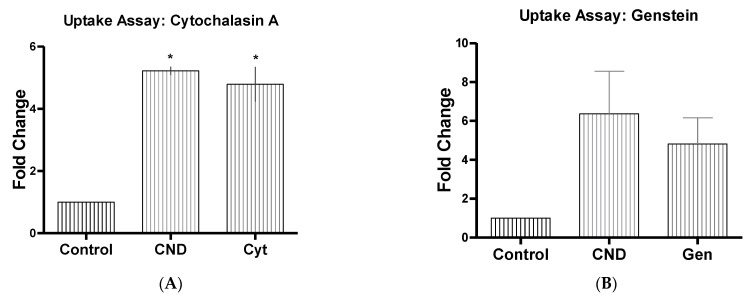

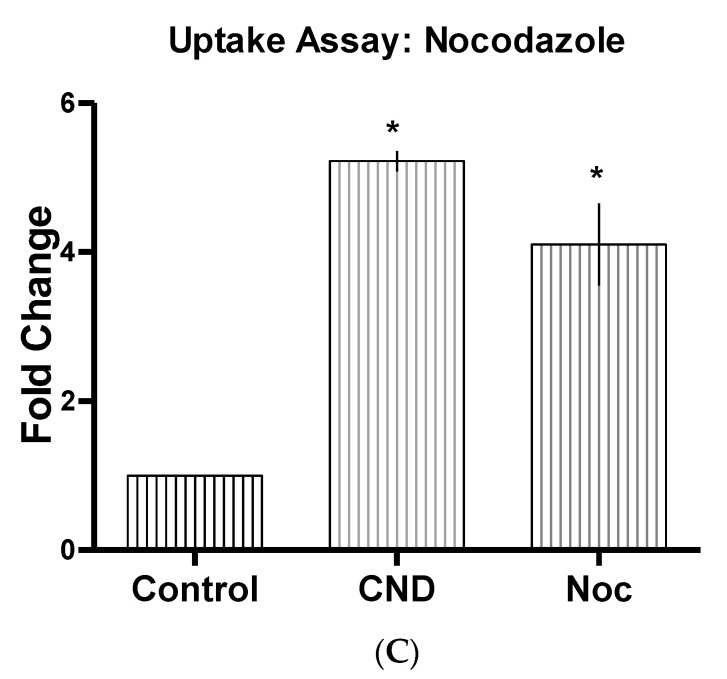
CND uptake with endocytosis disruptor. HMEC-1 cells were treated with inhibitor Cyt (**A**), Gen (**B**) and Noc (**C**) with concentrations described (Table 1), for 30 min. Cells were then washed two times with PBS and treated for 6 h with 0.1 mg/mL of CNDs. After CND treatment, cells were washed two times with PBS and removed. Cells’ fluorescence was checked using the Bio-Tek^®^ Synergy plate reader at 360/40 excitation and 460/40 emission. Endocytosis disruptors displayed did not show a significant change in fluorescent intensity. All data represent mean ± SEM. (n = 3, * *p* < 0.05 vs. Control).

Channel blocking inhibitors were also tested due to CND’s known ability for having a slight negative charge [33,34]. HMEC-1s were this time treated for 30 min with known channel blocking inhibitors at concentrations from previously completed research (Table 1). After the 30-min treatment, the 6-h CND treatment with 0.1 mg/mL began, and once treatment was completed, cells were washed two times with 1× PBS to ensure the removal of excess CNDs. Amlodipine (Figure 7C), a known calcium channel blocker, showed a significant increase in fluorescence intensity (*p* < 0.05). Further studies were completed using other channel blockers (Figure 8A–L), which all showed no significant changes in fluorescent intensity (*p* > 0.05). Other endocytosis disrupting inhibitors were also tested (Figure 9A–C) and demonstrated no significant change in fluorescence of CNDs (*p* > 0.05).

**Table 1:** Inhibitors used for uptake assay. List of channel blockers and endocytosis disruptors. Display of abbreviated name, concentrations used, and the function of the potential inhibitors.

Inhibitor Name	Abbrev	Concentrate	Function
4-Aminopyridine ~98%	4-AP	5 mM	Ion channel blocker (K^+^) [35]
Amiloride Hydrochloride Dihydrous	Amil	50 µM	Inhibits micropinocytosis: blocks Na^+^/H^+^ exchanger pump [36,37,38]
Amiodarone Hydrochloride	Amio	10 µM	Non-selective ion channel blocker [39]
Amlodipine	Aml	10 µM	Ion channel blocker (Ca^+^) [40]
Anthracene-9-Carboxilic Acid	Ant	100 µM	Ion channel blocker (Cl^−^) [41]
Barium Chloride Anhydrous	Ba	350 µM	Ion channel blocker (K^+^) [35]
Cesium Chloride, 99%	Cs	1 mM	Ion channel blocker (K^+^) [42]
Chlorpromazine HCL	Chl	10 µM	Suppresses clathrin disassembly [32,36]
Cobalt (II) Chloride	Co	2 mM	Ion channel blocker (Ca^+^) [43]
Copper Sulfate	Cu	100 µM	hAQP3 Aquaporins [44]
Cytochalasin A	Cyt	5 µg/mL	Actin disruptor [32]
Ebselen	Eb	15 µM	Inhibits mammalian H^+^, K^+^-ATPase [45]
Genstein	Gen	200 µM	Inhibits tyrosine kinase receptors [32]
Mercury Chloride	Hg	50 µM	hAQPI Aquaporins [44]
N-Phenlanthranilic Acid	N-Ph	0.1 mM	Ion channel blocker (Cl^−^) [46]
Niflumic Acid	Nif	10 µM	Ion channel blocker (Cl^−^)
Nocodazole	Noc	20 µM	Actin and microtubule disruptor [32]
Phenylglyoxal	Phen	100 µg	Selective inhibitor of phagocytosis [47]

## 4. Discussion

TNF-α is a known proinflammatory cytokine and can trigger a positive feedback loop, producing more TNF-α along with other proinflammatory cytokines, chemokines and adhesion molecules [6]. This study demonstrated that CNDs have the potential to reduce TNF-α induced inflammation in HMEC-1s by reducing the release of proinflammatory cytokines and chemokines. HMEC-1s were selected for this study [48]. This endothelial cell line that has been immortalized is commonly used for investigation of CVD due to their ability to retain many endothelial characteristics, such as the expression of ICAM and their ability to uptake LDL, and they have also been widely characterized [49]. There are currently no studies examining the effects of gene expression of phase II antioxidant enzymes during a co-treatment with TNF-α and CNDs. In our study, HO-1 relative gene expression saw a significant increase, demonstrating that CNDs are not only free radical scavengers but could potentially affect gene expression in genes related to phase II enzymes. Our studies also explored CND uptake routes by using the fluorescent characteristics of these nanoparticles. Compared with cells treated with CND only, two endocytosis disruptors and one channel blocker showed significant changes in CND uptake in endothelial cells. Furthermore, exploration of the cytotoxicity of CNDs was tested, and these nanoparticles did not appear to display toxic effects as assessed by MTT assay (Figure 2). Collectively, these finding suggests a potential anti-inflammatory action of CNDs against endothelial inflammation.

IL-8 is a proinflammatory cytokine that can cause an extremely rapid response from the immune system, and it has been shown to be highly expressed during cellular dysfunction of endothelial cells [50,51]. It has been shown in previous studies that when endothelial cells were treated with TNF-α, a dose-dependent increase in IL-8 gene expression was noticed [52]. ICAM is an adhesion molecule and is typically always present on the cell membrane of endothelial cells; however, during cellular stress, an influx in gene expression and appearance of the adhesion molecule are indicators of dysfunction [53,54]. The increase of this adhesion molecule is essential during the initiating process of atherosclerosis. Because of this molecule, monocytes and other leukocytes are able to bind to these distressed endothelial cells, and the transmigrational event occurs [55]. IL-1β is another proinflammatory cytokine that is responsible for increased vascular permeability of endothelial cells, while MCP-1 is a chemoattractant that is responsible for recruiting monocytes to the inflamed area [26,56]. The activation of these proinflammatory molecules is a biomarker for endothelial cell dysfunction and inflammation [26,50,52,53,54,56]. Our study, for the first time, has shown that CND treatment significantly suppressed TNF-α induced expression of IL-8, ICAM, and IL-1β, suggesting a potential anti-inflammatory action of CNDs against vascular dysfunction.

The mechanism by which CNDs reduces TNF-α induced inflammation remains unclear and is an area that requires further exploration. However, the ability of ROS scavenging by CNDs could be the mechanism that contributes to the pro-inflammatory properties based on two of the following reasons. Previous studies have demonstrated the capability of CNDs as antioxidants and scavengers of ROS. We have demonstrated that CNDs have the capability to be free radical scavengers through DPPH● testing. In this test, transferring of an electron either through oxidation or reduction can stabilize and reduce the absorbance reading. It was demonstrated that CNDs reduced the absorbance of DPPH in a dose-dependent manner as low as 0.02 mg/mL in the solution, demonstrating antioxidant, free radical scavenging potentials [24,57]. *Xanthine oxidoreductase* (XO) is present in most body tissues and fluids. Using the reaction carried out by XO reduces oxygen into a superoxide and lucigenin as the chemiluminescence probe; we further demonstrated CNDs scavenging ability of superoxide free radicals [24]. By way of Di-Chloro Di-Hydrofuran Fluorescein Di-Acetate (DCFH-DA) assay and NBT (Nitro Blue Tetrazolium) reduction assay, Das et al. showcased CNDs scavenging ability of hydroxyl and superoxide free radicals [20]. Altogether, these results denote the antioxidant propensity of CNDs and evidence their potential for biological utilization. Excess ROS and its subsequent oxidative stress are known to induce vascular inflammation [58,59]. An increase in cellular ROS was reported to be involved in the inflammatory effects of TNF-α on endothelial cells [21]. While ROS can occur naturally and the bulk of it for endothelial cells can come from the mitochondrial leakage, it has been reported that NADPH oxidase activation occurs with TNF-α [60,61]. NADPH oxidase is an enzyme responsible for facilitating the production of superoxide anions by removing an electron from NADPH and passing it to oxygen. This transfer of electrons causes a redox cycle to form and overproduction of ROS [62]. The literatures provided by this previous research support the possibility that CNDs have ROS scavenging activity and may be the explanation for the anti-inflammatory effects these nanoparticles have on lowering the TNF-α induced proinflammatory biomarkers in our study.

ROS can also be regulated by NQO1, Nrf2, GCLC, GR, AR, and HO-1 [27,28,29,31,63]. Our study investigated if this anti-inflammatory effect CNDs can be explained through changes in ROS modulator molecules in endothelial cells. However, our results showed no change in gene expression for the gene associated with the ROS modulator molecules except for HO-1. Interestingly, HO-1 gene expression had a significant increase with cells co-treated with CNDs at 0.3 mg/mL and TNF-α, and an increase like this due to CNDs has never been reported before. HO-1 has antioxidant, anti-inflammatory characteristics through cytoprotective qualities and is able to reduce apoptosis and reduce cellular distress [64,65]. Studies have shown that HO-1 gene expression at basal levels is typically low, but with an increase in those basal levels, endothelial cells expressed lower basal levels of proinflammatory cytokine IL-1β and adhesion molecule ICAM [64,66]. This is consistent with our findings of a decrease in gene expression of IL-1β and ICAM and an increase in HO-1 expression when HMEC-1s were co-treated with TNF-α and 0.3 mg/mL of CNDs. This reasoning behind the spike in HO-1 gene expression by CNDs is still unclear, but the Nrf2 pathway could explain the change [27,67]. Nrf2 is a transcription factor that is found in the cytosol of a cell and is sequestered by keap1. During cellular stress, ubiquitination occurs, allowing for the removal of keap1 and the transnuclear location of the Nrf2 transcription factor to enter and promote gene expression of antioxidants such as HO-1 [68]. Future studies will be needed to examine if the activation of the Nrf2 pathway occurs in HMEC-1s by CNDs, which could further explain the decrease in proinflammatory genes.

To further understand the action CNDs have on HMEC-1 cells, we studied the uptake of these nanoparticles. Diffusion, protein channels, or through endocytosis were the routes believed these nanoparticles could take; however, diffusion has been quickly ruled out due to the negative charge CNDs have [69]. Previous studies have been completed using different types of nanoparticles and different cell lines to examine potential routes of uptake. One study utilized HeLa, a cervical cancer cell line, and glycol chitosan nanoparticles. Different reagents were employed as potential inhibitors, to examine if they have any effect on the uptake of these nanoparticles. Chlorpromazine HCL was used due to the ability to inhibit clathrin-mediated endocytosis. Amiloride was used to inhibit micropinocytosis [36]. Results showed that these nanoparticles followed no particular route of endocytosis [36]. Another study used carboxylated polystyrene nanoparticles at either 40 nm or 200 nm in size in HeLa, A549 (lung carcinoma), and 1321N1 (brain astrocytoma) cell lines. Chlorpromazine HCL was used again along with genistein, which is an inhibitor of tyrosine kinase, cytochalasin A, an actin disruptor, and nocodazole, another chlathrin-mediated endocytosis [32]. Results from this study showed that particle size and cell type all played a part to the method of uptake [32]. In our study, we are exploring the effect of these inhibitors, along with other reagents that are known as endocytosis disruptors. Our results showed that phenylglyoxal, a known phagocytosis inhibitor [47], significantly reduced the fluorescence of CNDs. Chlorpromazine HCL, a clathrin disruptor, also reduced the fluorescence significantly. We began exploring other inhibitors, such as channel blockers, to see if other routes of uptake are possible due to the characteristics CNDs have [69]. After exploring our selected known channel blocking inhibitors, the results displayed that amlodipine, a calcium channel blocker [40], increased the fluorescence of CNDs. Previous studies completed on human umbilical vein endothelial cells (HUVEC) suggested that when a build-up of calcium occurs internally within endothelial cells, this stimulates an increase in endocytosis events [70]. Our results showed that phenylglyoxal and chlorpromazine significantly decreased CND uptake, while amlodipine increased it, supporting that CNDs route of uptake might not only be through endocytosis.

In summary, CNDs reduce TNF-α-mediated endothelial inflammation in HMEC-1 cells. Our results showed CNDs did not possess cytotoxic characteristics. Furthermore, our study was able to narrow down potential routes of uptake for CNDs into HMEC-1s, showing that endocytosis is indeed a route of entry; however, it might not be the only one. These findings provide insights on the interaction CNDs and endothelial cells undergoing TNF-α induced cellular inflammation.

## Data Availability

The data used to support the findings of this study are available from the corresponding author upon request.
